# Japanese Physicians

**Published:** 1860-07

**Authors:** 


THE JAPANESE PHYSICIANS.—During the recent visit of the Japanese Embassy to this city, we had several opportunities of visiting the physicians, and elicited from them a slight amount of information in regard to the state of medicine in Yedo. Their knowledge has been in great part derived from the Dutch, and we are sorry to state that the physicians that we saw, and who may be taken as fair specimens of their class, did not strike us as having much acquaintance with any of the departments of medicine or surgery. The lateral operation of lithotomy, which they saw performed by the senior editor, was altogether unknown to them, but they stated that the high operation was performed in Japan, the practice of which was taught them by the Dutch. They were much interested in the administration and the effects of ether, and will take a supply of it with them on their return to their native country, with full directions as to its preparation. Amputation is not practiced by them, nor did they seem to know anything of the ligature as a means of arresting hemorrhage. They perform the ordinary operation for hare-lip, and we saw one of their servants who had been treated for this affection with perfect success. The operation of catheterism is familiar to them, and they employ the silver catheter and gum bougie. The trocar, which is somewhat different from our own, is used by them for tapping hydroceles and ascitic abdomens. They have no experience in the surgery of the eye, as in fact is the case with all the larger operations. They are perfectly conversant with the temporal, carotid, and radial pulse, and recognize the heart as the prime source of the circulation. Beyond this, the whole subject of the circulation is unknown to them, and we frequently saw a number of the servants, as well as the doctors, apply three or four small moxas to their legs in order to promote the free circulation of the blood. Vaccination was introduced into Japan about six years ago, and it was formerly the custom to insert the virus in the mucous membrane of the nose; but at the present time the operation is performed on the arm. The operations which they saw performed during their stay in this city were, amputation at the hip and of the forearm, by Professor Pancoast, and the lateral operation of lithotomy, by Professor Gross. In the latter case ether was administered by Dr. Morton, of Boston.

